# Hemolytic Anemia in Liver Disease: A Case of Spur Cell Anemia

**DOI:** 10.7759/cureus.106340

**Published:** 2026-04-02

**Authors:** Hajar El Amri, Asmitha P Reddy, Waqas Azhar, Baqai Junaid

**Affiliations:** 1 Internal Medicine, Southern Illinois University School of Medicine, Springfield, USA; 2 Hematology/Oncology, Southern Illinois University School of Medicine, Springfield, USA; 3 Pathology and Laboratory Medicine, Springfield Memorial Hospital, Springfield, USA

**Keywords:** alcohol-related cirrhosis, disseminated intravascular coagulation (dic), hemolytic anemia, liver disease, spurr cell anemia

## Abstract

Spur cell anemia (SCA) is a rare but severe form of acquired hemolytic anemia seen in advanced cirrhosis and carries a poor prognosis in the absence of liver transplantation. We report the case of a middle-aged man with alcohol-related cirrhosis who developed profound anemia requiring recurrent transfusions. An extensive workup was performed to rule out other causes of anemia, including gastrointestinal bleeding, microangiopathic hemolytic anemia, and autoimmune hemolytic anemia. Peripheral smear confirmed the presence of acanthocytes, consistent with SCA. Given that he was not a transplant candidate, supportive transfusions were provided, but his condition deteriorated rapidly, and he died within one month of diagnosis. This case highlights the aggressive course of SCA in non-transplant candidates and underscores the need for further research into alternative therapies.

## Introduction

Spur cell anemia (SCA) is an uncommon, acquired form of hemolytic anemia that occurs in the setting of advanced liver disease, most frequently alcoholic cirrhosis. It is defined by the presence of acanthocytes, erythrocytes with irregularly spaced spicules on the peripheral smear. These projections are variable in length and distribution and must be distinguished from echinocytes (burr cells), which display uniformly spaced membrane projections often related to artifact or uremia, as well as schistocytes, which represent fragmented erythrocytes seen in microangiopathic hemolytic processes. The rigidity of acanthocytes predisposes them to premature sequestration and destruction in the spleen, resulting in severe hemolysis [1].

Clinically, SCA is characterized by rapidly progressive anemia, jaundice, and transfusion dependence. The condition portends a poor prognosis and is considered a marker of end-stage liver disease [2]. Because it is relatively rare and often underrecognized, SCA may be misattributed to other causes of anemia in cirrhotics, such as gastrointestinal bleeding, nutritional deficiencies, or bone marrow suppression. Recognition is therefore crucial, as it signifies advanced disease and influences both prognostication and transplant evaluation.

Here, we describe a case of SCA in a patient with decompensated alcoholic cirrhosis who experienced rapid clinical decline after diagnosis and died within 30 days, highlighting the aggressive nature of this entity and the importance of timely recognition.

## Case presentation

A 51-year-old man with decompensated alcohol-associated cirrhosis (Child-Pugh class C, Model for End-Stage Liver Disease (MELD) score of 27), complicated by prior hepatic encephalopathy, presented with new-onset jaundice. He had no history of hereditary hemolytic anemia and reported continued alcohol use a few days before admission.

On arrival, he was alert and oriented and denied hematochezia or melena. Examination revealed scleral icterus, jaundice, and non-tender abdominal distension. Laboratory evaluation demonstrated acute worsening of chronic anemia with a hemoglobin of 6.5 g/dL (baseline 11-12 g/dL) and total bilirubin of 19.8 mg/dL (Table 1). CT angiography of the abdomen and pelvis showed no active bleeding. He was transfused with packed red blood cells (pRBCs), and diagnostic paracentesis revealed no spontaneous bacterial peritonitis. Esophagogastroduodenoscopy showed grade I esophageal varices and portal hypertensive gastropathy without bleeding. Despite transfusions, hemoglobin remained persistently low.

**Table 1:** Laboratory values during each admission

Laboratory test	First hospitalization	Second hospitalization	Last hospitalization	Reference range
Hemoglobin (g/dL)	6.5	4.7	3.7	12.0-16.0
Hematocrit (%)	18%	13%	10%	36-46%
Red blood cell count (RBC) ×10⁶/µL (million cells per microliter)	1.67	1.24	0.85	4.7-6.1
White blood cell count (WBC) ×10³/µL (thousand cells per microliter)	4.5	7.8	14.5	3.4-9.4
Platelet count ×10³/µL (thousand cells per microliter)	103	55	69	150-450
Total bilirubin (mg/dL)	19.8	18.7	24.3	0.1-1.2
Direct bilirubin (mg/dL)	4.5	3.9	5.8	0.0-0.3
Aspartate aminotransferase (AST) (U/L)	71	66	58	10-40
Alanine aminotransferase (ALT) (U/L)	25	31	35	7-56
Lactate dehydrogenase (LDH) (U/L)	399	329	410	140-280
Haptoglobin (mg/dL)	<30	<30	<30	30-200
Albumin (g/dL)	4.2	3.1	3.4	3.5-5.0
International normalized ratio (INR)	2.2	2.8	2.4	0.8-1.2
Activated partial thromboplastin time (APTT) (seconds)	40.1	41.5	37.6	25-35
Fibrinogen (mg/dL)	111	99	119	200-400

Further testing revealed elevated lactate dehydrogenase (LDH), indirect hyperbilirubinemia, and undetectable haptoglobin, consistent with hemolysis. Reticulocyte count (167.3 x 10³cells/µL) was inappropriately low (reticulocyte production index of 1.5), suggesting impaired marrow response. Vitamin B12 and folate levels were normal. The peripheral smear initially showed normocytic anemia with occasional fragmented cells. Direct antiglobulin testing was negative. Coagulation studies demonstrated prolonged PT (prothrombin time), aPTT, and INR with low fibrinogen and elevated fibrin split products. These findings were consistent with cirrhosis-associated coagulopathy or acute disseminated intravascular coagulation (DIC). However, the International Society on Thrombosis and Haemostasis (ISTH) DIC score was <5, making overt DIC unlikely, particularly in the absence of active bleeding or thrombosis. His anemia was therefore attributed to decompensated cirrhosis with hypersplenism, and he was discharged following clinical stabilization.

One week later, he was readmitted with hemoglobin 4.7 g/dL and acute kidney injury, which improved with transfusion. Thrombocytopenia was unchanged from baseline, but concern remained for microangiopathic hemolytic anemia. ADAMTS13 activity was borderline low with normal inhibitor levels (ADAMTS13 inhibitor <0.4 BU/mL), excluding thrombotic thrombocytopenic purpura. Testing for antiphospholipid antibodies was negative, and paroxysmal nocturnal hemoglobinuria was excluded by flow cytometry. Bone marrow biopsy revealed a hypercellular marrow with trilineage hyperplasia and no evidence of myelodysplastic or myeloproliferative disorder.

**Figure 1:**
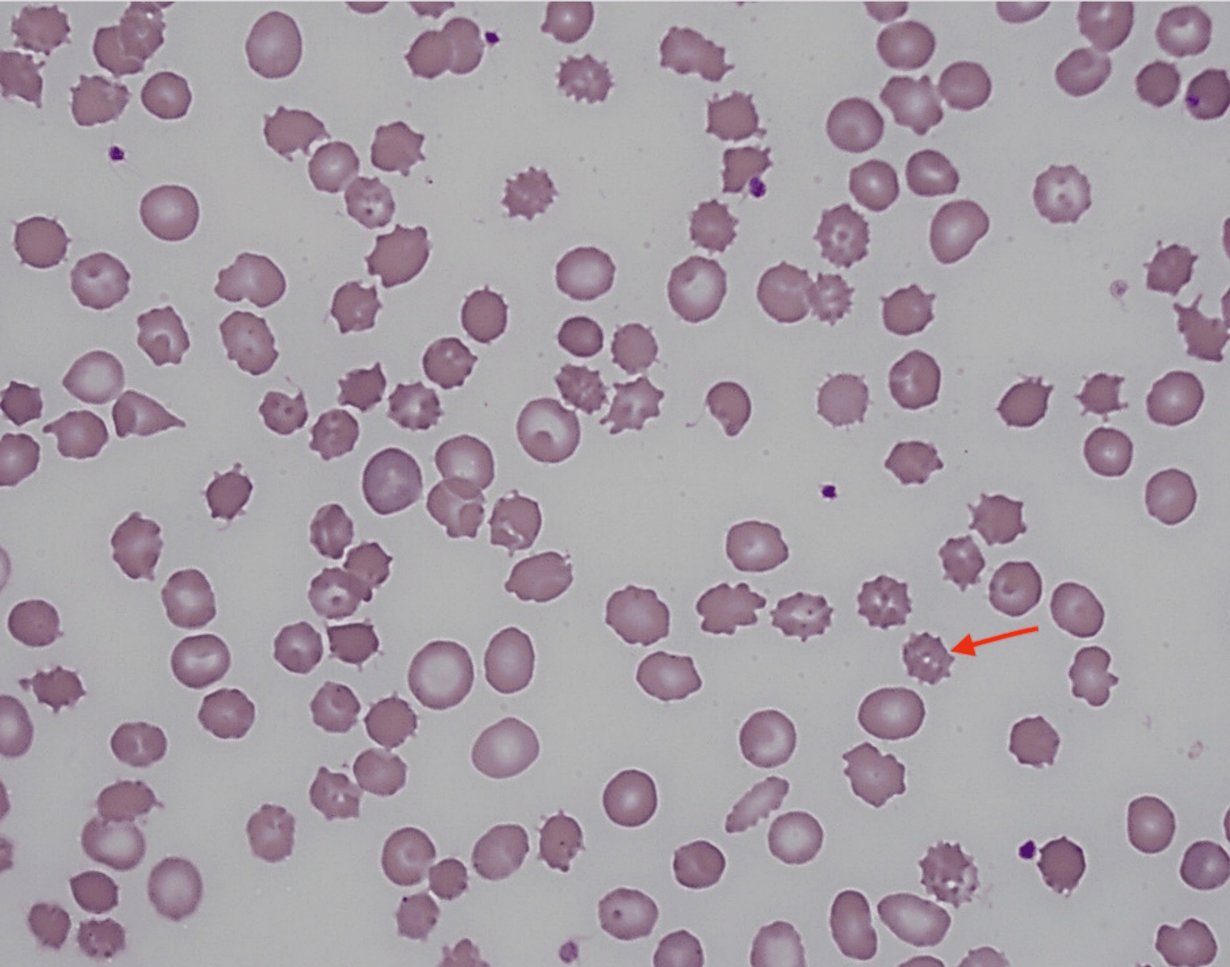
Peripheral blood smear (Wright–Giemsa stain, 1000× magnification) showing multiple acanthocytes (red arrow).

A repeat peripheral blood smear was obtained (Figure 1), and prior smear review revealed numerous acanthocytes estimated at 6% (“sphero-acanthocytes”). In the setting of a negative extensive hemolytic workup, the patient was diagnosed with SCA. Given the poor prognosis associated with this condition and the fact that liver transplantation is the only curative therapy, the patient was referred to two transplant centers. He was not eligible for inpatient liver transplantation because of ongoing alcohol consumption, confirmed by an elevated phosphatidylethanol level of 69 ng/mL (abstinence <20 ng/mL). He later admitted to alcohol consumption three days after his initial hospital discharge. He was discharged with close outpatient follow-up.

He subsequently returned a third time to the hospital with profound weakness, hemoglobin 3.7 g/dL, and thrombocytopenia (Table 1), prompting multiple transfusions. Despite aggressive supportive care, his clinical course was marked by rapid deterioration. He developed multifactorial shock, including cardiac tamponade, refractory acute kidney injury with anuria, worsening metabolic acidosis, hyperkalemia, and progressive multiorgan failure. His hospitalization was further complicated by bleeding and thrombotic events, including recurrent hematomas and deep vein thrombosis, secondary to acute disseminated intravascular coagulation.

Across these hospitalizations, the patient demonstrated transfusion-refractory hemolysis. He was repeatedly transfused and discharged with hemoglobin levels above 7 g/dL, yet was consistently readmitted with worsening anemia. Laboratory trends showed persistently elevated hemolysis markers, including increased lactate dehydrogenase, undetectable haptoglobin, and rising bilirubin (Table 1).

Given his ineligibility for liver transplantation, the only curative option for SCA in the setting of advanced cirrhosis, the family elected to transition to comfort-focused care. He ultimately passed away 32 days after his initial presentation.

## Discussion

SCA is a rare but severe acquired hemolytic anemia that occurs in the setting of advanced liver disease, including both alcoholic cirrhosis and nonalcoholic steatohepatitis (NASH)-related cirrhosis. It is distinct from the more common hypersplenism-related hemolysis observed in chronic liver disease and is characterized by the presence of acanthocytes (erythrocytes with multiple irregularly spaced membrane spicules) on peripheral blood smear, reflecting profound alterations in red blood cell membrane composition and function. These cells must be distinguished from echinocytes (burr cells), which display uniformly spaced membrane projections often related to artifact or uremia, as well as schistocytes, which represent fragmented erythrocytes seen in microangiopathic hemolytic processes [1]. Importantly, SCA reflects advanced hepatic synthetic dysfunction and should be regarded as a hematologic manifestation of end-stage liver disease and a marker of poor prognosis rather than an isolated red cell disorder.

The pathogenesis of SCA is driven by abnormalities in red cell membrane lipid composition, particularly an increased cholesterol-to-phospholipid ratio. Excess membrane cholesterol reduces erythrocyte deformability and membrane fluidity, rendering cells more susceptible to mechanical injury and splenic sequestration. Impaired hepatic synthesis of lipoproteins and lipolytic enzymes, including lecithin-cholesterol acyltransferase and lipase, further disrupts membrane homeostasis. Nutritional deficiencies frequently seen in advanced cirrhosis compound these abnormalities, collectively leading to markedly reduced erythrocyte survival. When superimposed on hypersplenism, these processes result in the profound hemolysis characteristic of SCA [2].

Clinically, patients often present with fatigue, pallor, and jaundice, accompanied by laboratory evidence of hemolysis, including elevated lactate dehydrogenase and indirect hyperbilirubinemia. Transfusion dependence is common and portends a poor prognosis. In one study, transfusion dependence was associated with a significantly increased 90-day mortality risk (odds ratio 9.14; 95% CI, 2.46-34.00) [3]. SCA is generally diagnosed when acanthocytes comprise more than 5% of circulating erythrocytes in the presence of anemia (hemoglobin <10 g/dL). Although the overall incidence remains uncertain, Sousa et al. reported SCA in 4.13% of 339 patients with cirrhosis [4]. Importantly, the proportion of spur cells correlates with disease severity; patients with >5% acanthocytes demonstrate worse liver dysfunction and lower short-term survival compared with those with 1-4% [5].

Differentiating SCA from other causes of anemia in cirrhosis is clinically essential. In cirrhotic patients presenting with an acute hemoglobin decline, upper gastrointestinal bleeding must be promptly considered and excluded, given its frequency and clinical urgency. Gastrointestinal bleeding typically presents with acute anemia without significant reticulocytosis unless chronic. Hypersplenism, in contrast, produces cytopenias primarily through splenic sequestration, often resulting in thrombocytopenia and leukopenia alongside anemia. Although mild extravascular hemolysis may occur in the setting of splenomegaly, it is generally limited and does not account for the severe hemolysis or marked acanthocytosis seen in SCA.

In cases of severe hemolytic anemia, additional etiologies must be excluded, particularly microangiopathic hemolytic anemia in the context of disseminated intravascular coagulation or thrombotic thrombocytopenic purpura, which are characterized by schistocytosis, worsening baseline thrombocytopenia in cirrhotics, and consumptive coagulopathy rather than spur cells. Autoimmune hemolytic anemia should also be considered and is typically identified by a positive direct antiglobulin test and spherocytosis on peripheral smear. Careful integration of smear morphology, hemolysis laboratories, and coagulation studies is therefore essential to accurately differentiate these entities from SCA.

Liver transplantation remains the only definitive therapy for SCA, leading to rapid resolution of hemolysis and disappearance of spur cells following restoration of hepatic function [3,4]. Early recognition of SCA is therefore critical, as it may prompt timely transplant evaluation before further hepatic decompensation or relapse, when feasible. Unfortunately, our patient was not a transplant candidate due to ongoing alcohol use and experienced a rapid clinical decline, dying within one month of diagnosis. This course aligns with prior reports demonstrating three-month survival rates of approximately 33% among non-transplant candidates with SCA [3].

Given the dismal prognosis in patients ineligible for transplantation, several alternative therapies have been explored in isolated reports. Combination therapy with flunarizine, pentoxifylline, and cholestyramine led to the resolution of spur cells and lipid abnormalities in a single case, though concurrent alcohol cessation limited attribution of benefit to pharmacologic therapy alone [6]. Polyunsaturated phosphatidylcholine infusion (2 g daily for 5 days) has also been associated with regression of spur cells, although durability and survival impact remain unknown [7]. High-dose corticosteroids have been reported to stabilize hemoglobin and reduce acanthocytosis in a patient with NASH cirrhosis [8]. Splenectomy has demonstrated reductions in hemolysis but carries substantial perioperative risk, including bleeding and mortality [9]. Despite these reports, no large-scale studies have validated these interventions, and their clinical benefit remains uncertain.

## Conclusions

SCA is an underrecognized complication of advanced cirrhosis that is frequently overlooked in favor of more common etiologies of anemia, including hypersplenism, gastrointestinal bleeding, and bone marrow suppression. Failure to identify this entity may delay transplant evaluation and risk missing a critical window for intervention. Careful examination of the peripheral smear should therefore be routinely incorporated into the diagnostic workup of cirrhotic patients with unexplained anemia, particularly in the presence of laboratory evidence of hemolysis. This case underscores the importance of early recognition of SCA as a hematologic marker of end-stage liver disease with well-established adverse prognostic implications independent of this single report. However, as highlighted by this case, timely recognition alone does not guarantee access to the transplant window. Although liver transplantation remains the only intervention shown to improve outcomes, our patient’s inability to undergo transplantation was not attributable to delayed diagnosis, referral, or transfer, but rather to ongoing alcohol use, confirmed by an elevated phosphatidylethanol level, which precluded transplant eligibility. This emphasizes that early diagnosis must be paired with strict sobriety monitoring and fulfillment of transplant candidacy criteria to meaningfully impact outcomes.
